# CNTs Intercalated LDH Composite Membrane for Water Purification with High Permeance

**DOI:** 10.3390/nano12010059

**Published:** 2021-12-26

**Authors:** Qian Li, Peng Song, Yuye Yang, Yan Li, Naixin Wang, Quanfu An

**Affiliations:** Beijing Key Laboratory for Green Catalysis and Separation, Department of Environmental and Chemical Engineering, Beijing University of Technology, Beijing 100124, China; liqian2502@163.com (Q.L.); yangyy0524@163.com (Y.Y.); nayil@emails.bjut.edu.cn (Y.L.); wangnx@bjut.edu.cn (N.W.); anqf@bjut.edu.cn (Q.A.)

**Keywords:** carbon nanotube, layered double hydroxide, vacuum-assisted assembly, composite membrane, water purification

## Abstract

The pursuit of improved water purification technology has motivated extensive research on novel membrane materials to be carried out. In this paper, one-dimensional carboxylated carbon nanotubes (CNTs) were intercalated into the interlayer space of layered double hydroxide (LDH) to form a composite membrane for water purification. The CNTs/LDH laminates were deposited on the surface of the hydrolyzed polyacrylonitrile (PAN) ultrafiltration membrane through a vacuum-assisted assembly strategy. Based on the characterization of the morphology and structure of the CNTs/LDH composite membrane, it was found that the intercalation of CNT created more mass transfer channels for water molecules. Moreover, the permeance of the CNTs/LDH membrane was improved by more than 50% due to the low friction and rapid flow of water molecules in the CNT tubes. Additionally, the influence of preparation conditions on the separation performance was investigated using Evans blue (EB). Optimized fabrication conditions were given (the concentration of CoAl-LDH was 0.1 g/L and the weight ratio of CNTs was 2 wt.%). Next, the separation performances of the prepared CNTs/LDH composite membrane were evaluated using both single and mixed dye solutions. The results showed that the composite membrane obtained possessed a retention of 98% with a permeance of 2600 kg/(m^2^·h·MPa) for EB, which was improved by 36% compared with the pristine LDH composite membrane. Moreover, the stability of the CNTs/LDH composite membrane was investigated in 100 h with no obvious permeance drop (less than 13%), which exhibited its great potential in water purification.

## 1. Introduction

The search to improve the quality of water resources has motivated researchers to develop versatile wastewater treatment techniques. Adsorption, reductive and oxidative processes, phytoremediation, and membrane separation are some of the main techniques used for the removal of organic pollutants [1]. Among these techniques, membrane separation has attracted a great deal of attention, especially for the removal of small organic molecules and high-valence inorganic salts [2,3,4,5]. Studies of membranes have aided in the development of various research fields, including wastewater treatment [6,7,8,9], the pharmaceutical industry [10,11,12], food processing [13,14], and bioengineering [15,16]. During the separation process, both steric hindrance and the electrostatic repulsion effect determine the specific performance of a membrane [17]. Obviously, the interaction of both mechanisms requires the ingenious design of the membrane structure; the corresponding membrane materials are therefore vital for the construction of separation layers [18,19,20].

The development of organic–inorganic composite membranes integrates the benefits of both organic and inorganic membranes for the achievement of excellent separation performances. Organic materials, including polyamide [21], polyimide [22], polyvinyl alcohol [23], chitosan [24], and cellulose acetate [25], have been extensively used for the preparation of membranes. However, the separation performance of these polymeric membranes, and especially their permeance, needs to be further improved [26]. Two-dimensional (2D) materials—e.g., graphene oxide (GO) [27], molybdenum disulfide (MoS_2_) [28], layered double hydroxide (LDH) [29], and MXene [30]—are thus widely employed in membrane fabrication considering their superior water transport and ion selectivity properties. Among all these 2D materials, LDH possesses the advantages of tunable layer spacing and versatile surface properties and microstructures via its combinations of positively charged laminates and interlayer anions [31,32]. Such features have facilitated the extensive application of LDH in composite membranes. For instance, the introduction of ZnAl-LDH into a polyamide nanofiltration membrane allows the fabrication of denser polyamide layers with more leaf-like folds and a higher hydrophilicity and water permeability [33]. Nevertheless, stacking 2D nanomaterials is not favorable for the fabrication of membranes due to defects on the membrane surface.

Comparatively, exfoliated LDH nanosheets have the advantages of a thin layer thickness, large specific surface area, and superior integration of nanosheets, which facilitates the fabrication of defect-free hybrid membranes [34,35]. For example, Dong et al. reported a Co(OH)_2_-LDH hybrid membrane with an improved stability against salt and rejection of organic molecules [36]. A flux of 17.0 L/(m^2^·h) with a rejection of inorganic salts (>91%) and organic dyes (>98%) was achieved. Additionally, a thin-film composite membrane employing MgAl-LDH nanosheets and sulfonated polysulfone was reported by Lu et al. for a minimum leaching of sulfonated polysulfone with an enhancement of membrane properties [37]. Moreover, an exfoliated hydrotalcite/graphene oxide (EHT/GO) hybrid nanosheet membrane was synthesized by Wang et al.; it could achieve an increase in permeance from 7.37 to 26 L/(m^2^·h) with a rejection of MgCl_2_ of up to 97% [38]. Thus, exfoliated LDH nanosheets can be used as a robust assembly unit for promoting water transport. According to the structure of LDH, intercalating material between positively charged laminates also determines the properties of a membrane. In this study, carbon nanotubes (CNTs) were chosen as an intercalation material with which to build the membrane due to their large specific surface area, small pore size, and excellent mechanical properties [39]. Moreover, CNTs have a smooth graphitic surface and quick adsorption–desorption mechanism, which promotes the transport of water molecules and alleviates the development of membrane fouling [39]. For instance, a multiwalled carbon nanotube (MWCNTs)/polyethersulfone nanocomposite membrane was fabricated by Vatanpour et al., and the hydrophilicity, pure water flux, and antifouling properties were all significantly improved via the addition of MWCNTs [40]. Additionally, Han et al. reported a high-flux nanofiltration membrane fabricated by the synergistic assembling of graphene oxide and MWCNTs. The permeance of the obtained membrane was increased by more than two times and the excellent antifouling properties were verified by serum albumin (BSA) and humic acid (HA) [41].

In this paper, the development of a carboxylated CNT intercalated LDH composite membrane is reported for use in water purification with a high permeance. The membrane structure and properties were systemically investigated by XRD, SEM, and AFM. Based on the characterization results, we concluded that the CNTs were successfully intercalated into the CoAl-LDH laminates and the fabrication conditions, including the CNT weigh ratio and CoAl-LDH solution concentrations, were investigated. Next, the separation performances were examined using six single dye solutions, including eriochrome black T (EBT), Congo red (CR), Evans blue (EB), rhodamine B (RhB), methylene blue (MB), and methyl orange (MO), and different dye mixtures (EB/MO, MB/CR, EBT/MO, and RhB/EB). Moreover, a long-term stability test of the obtained membrane was carried out using humic acid (HA) to further evaluate the potential industrial application of the CNTs/LDH composite membrane.

## 2. Materials and Methods

### 2.1. Materials

Polyacrylonitrile (PAN) ultrafiltration membrane with a molecular weight cutoff (MWCO) of 50,000 Da was supplied by Zhongkeruiyang Membrane Engineering & Technology Co., Ltd. (Beijing, China). Cobalt (II) nitrate hexahydrate, aluminum nitrate nonahydrate, urea, sodium hydroxide, ethylene glycol (EG), ethanol, acetone, HA, EBT, CR, EB, RhB, MB, and MO were all obtained from Beijing Chemical Factory. The molecular structures and weights of the dyes used in this paper are summarized in Table 1. Carboxyl-functionalized multi-walled carbon nanotubes (MWCNTs-COOH) were purchased from Beijing Inno Chem Science & Technology Co., Ltd (Beijing, China). The MWCNTs-COOH had a length of 10–30 μm, an outer diameter of 20–30 nm, and an inner diameter of 5–10 nm. All chemicals were used without further purification.

### 2.2. Preparation of CNTs/LDH Composite Membranes

A pretreatment was carried out on PAN membrane to boost the interaction between the synthesized composite and the substrate. The PAN substrate was soaked in an ethanol–water solution (ethanol = 30 wt.%) for 2 h to remove all residues. Then, the membrane was filtrated in a filter flask until no bubbles were observed. After that, the PAN membrane was hydrolyzed in a 2 mol/L NaOH solution at 65 °C for 30 min and then cleaned with deionized water. The cleaned membrane was finally kept in deionized water for further use.

**Figure 1:**
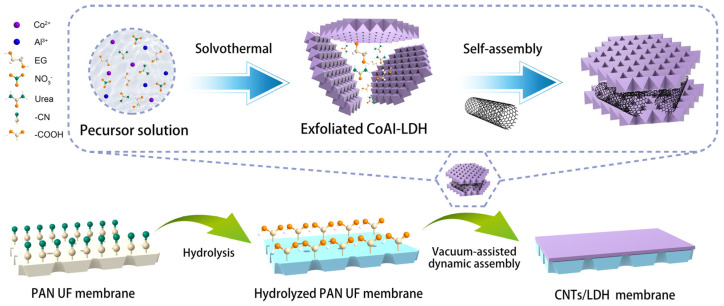
Schematic illustration of the preparation of the CNTs/LDH composite membrane.

The synthesis of exfoliated CoAl-LDH nanosheets has been described elsewhere [42]. The obtained nanosheets were dispersed in water with 10 min of sonification to obtain a colloidal solution for further use. The CNT dispersed solution was prepared by adding 0.05 g of MWCNTs-COOH to 100 mL of degassed water, followed with ultrasonication for 30 min. The mixture was then centrifuged at 1000 rpm for 15 min and only the supernatant was collected. The aforementioned supernatant solution was subsequently dried in an oven and the CNT powder was obtained for the fabrication of composite membranes. Afterwards, a CNTs/LDH mixture was prepared by doping different amounts of CNT powder (1–5 wt.%) directly in CoAl-LDH dispersions with 10 min of ultrasonication. Then, 100 mL pf CNTs/LDH solution was filtrated on the hydrolyzed PAN substrate to obtain the CNTs/LDH composite membranes via a vacuum-assisted assembly method. For comparison, the CNT membrane and CoAl-LDH membrane were prepared using the same methodology. A detailed schematic illustration of the fabrication of the composite membrane is given in Figure 1.

### 2.3. Membrane Characterizations

The X-ray powder diffraction (XRD) patterns were recorded in the 2θ range of 5–20° with a scan step of 0.5° using a Cu-Kα radiation under 40 kV and 40 mA (Bruker D8 Advance diffractometer, Bruker, Karlsruhe, Germany). The surface and cross-sectional morphologies and structures of the CNT membrane, CoAl-LDH membrane, and CNTs/LDH composite membrane were observed using scanning electron microscopy (SEM) (SU-8020, Hitachi, Tokyo, Japan). The surface roughness of the fabricated membrane was characterized by atomic force microscopy (AFM) (Pico ScanTM 2500, Tempe, AZ, USA).

### 2.4. Evaluation of Membrane Separation Performance

The obtained membranes were evaluated by a laboratory-made separation system, which had an effective membrane area of 7.1 cm^2^. Both the anionic dyes (EBT, EB, and CR) and cationic dyes (MO, MB, and RhB) were fixed at 0.1 g/L, while the humic acid was set as 0.01 g/L for the separation experiment. The filtration pressure was kept at 0.1 MPa. As the main characteristic parameters used to evaluate the membrane performance, membrane permeance (*F*) and retention (*R*) were employed here for the characterization of the membrane:

(1) $$F=\frac{M}{AtP}$$

where *M* is the weight of permeate collected within the filtration time *t*, *A* is the effective membrane area, and *P* is the operation pressure.

(2) $$R=\left(1-\frac{C_{p}}{C_{f}}\right)\times 100\%$$

where *C_p_* is the solute concentration in the permeate and *C_f_* is the solute concentration in the feed. A UV–vis spectrophotometer (UV-6100) was employed to analyze the feed and permeate concentrations.

## 3. Results and Discussion

### 3.1. CNTs/LDH Membrane Characterization

**Figure 2:**
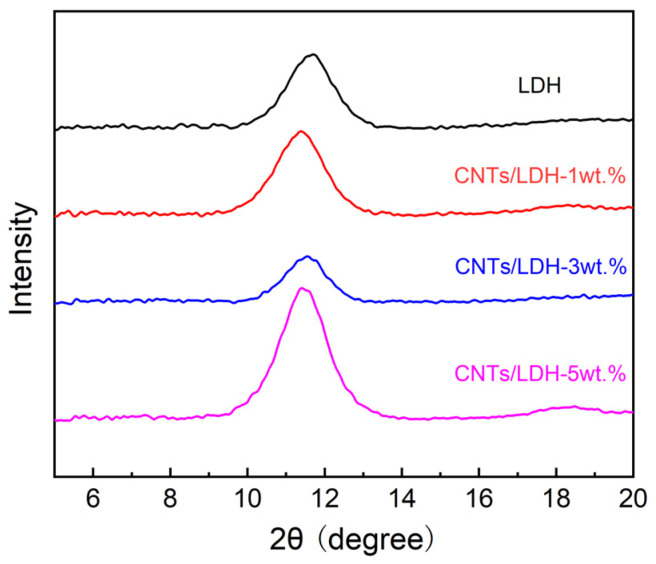
The XRD patterns of the CoAl-LDH membrane and CNTs/LDH membranes with different mass fractions of CNTs (1 wt.%–5 wt.%).

The structures of the obtained CNTs/LDH composite membranes were initially characterized by XRD. As illustrated in Figure 2, the characteristic peak at 11.7° corresponded to the (003) crystal plane of CoAl-LDH, clearly verifying the formation of the synthesized LDH on the membrane. The characteristic peak representing the (003) crystal plane of LDH shifted to 11.4° when CNTs were introduced. Hence, such shifting behavior confirmed the successful intercalation of CNTs in the interlayer of LDH, which resulted in an increase in the interlayer space from 0.755 to 0.77 nm through the Bragg equation. Moreover, no shifting of the corresponding characteristic peak was observed along with the addition of CNTs up to 5 wt.%, thus demonstrating the invariable interlayer spacing of CNTs/LDH.

**Figure 3:**
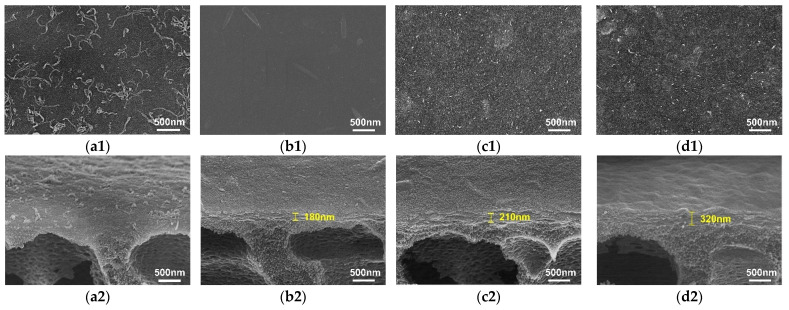
Surface and cross-sectional SEM images of (**a1,a2**) the 3 ppm CNTs membrane, (**b1,b2**) CoAl-LDH membrane, (**c1,c2**) CNTs/LDH-3 wt.% membrane, and (**d1,d2**) CNTs/LDH-5 wt.% membrane.

The CNTs/LDH membrane was further characterized by SEM. The surface and cross-sectional morphologies of the CNT membrane, LDH membrane, and CNTs/LDH membranes with different weight ratios of CNT are given in Figure 3. The surface and cross-sectional morphologies of the CNT membrane are revealed in Figure 3a1,a2, respectively. An obvious agglomeration of CNTs was observed on the surface of the membrane, as well as plenty of pores on the membrane surface. Additionally, no evident separation layers on the substrate are demonstrated in Figure 3a2, which further verifies the failure of the compact separation layer with only CNTs. Comparatively, the substrate covered with LDH showed a compact separation layer without defects on the substrate, as shown in Figure 3b1, which apparently stemmed from the deposition of smooth and compact laminated structures. The obtained separation layer thickness was 180 nm, as depicted in Figure 3b2. The morphology of the CNTs/LDH-3 wt.% membrane is given in Figure 3c. Clearly, CNTs were intercalated between the LDH nanosheets via electrostatic force and the obtained self-assembly separation layer offered rapid transport channels for water molecules. Additionally, the successful intercalation resulted in an enlarged separation layer of 210 nm, as shown in Figure 3c2. Along with the increase in the amount of CNTs to 5 wt.%, the resultant membrane had no defects, as illustrated in Figure 3d1. Moreover, the thickness of the compact separation layer increased to 320 nm, clearly indicating the uniform distribution of CNTs in the intercalation behavior between LDH and CNTs.

**Figure 4:**
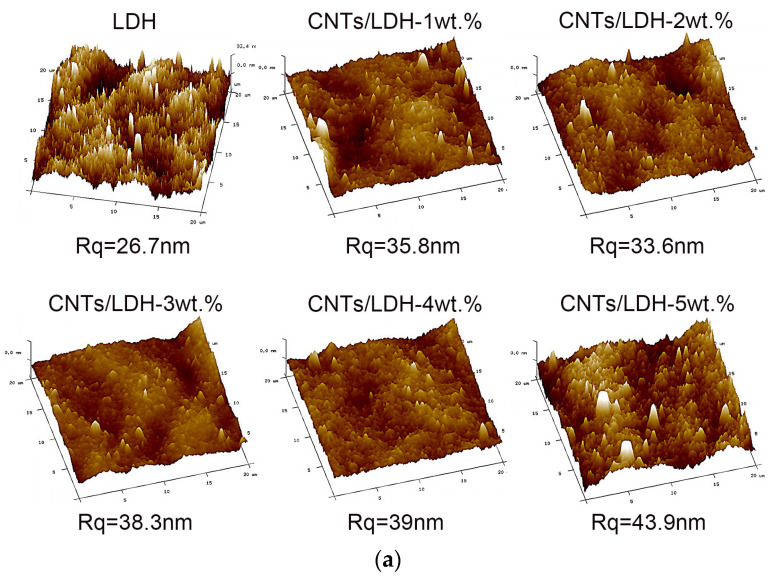

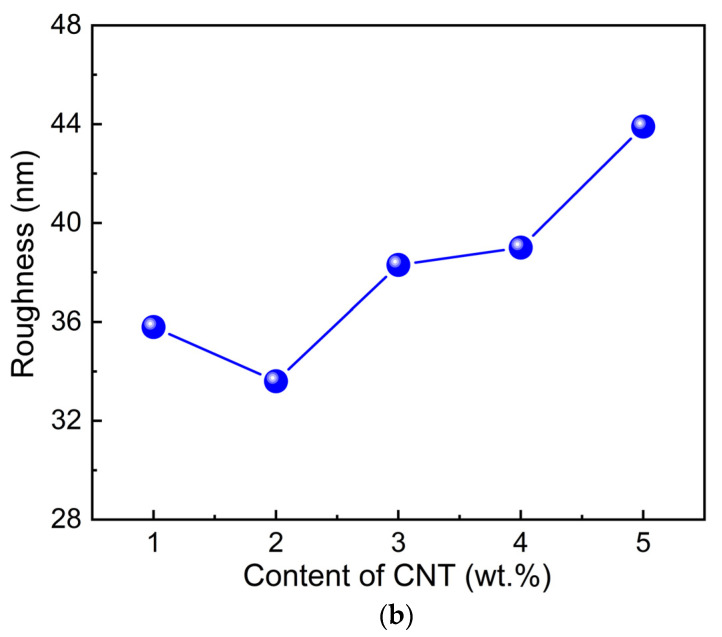
(**a**) AFM images of LDH and CNTs/LDH membranes; (**b**) surface roughness of CNTs/LDH composite membranes with different CNT contents (1 wt.%–5 wt.%).

Next, the surface roughness of the CNTs/LDH membrane was investigated using AFM, as shown in Figure 4a,b. As expected, the Rq of the pristine LDH membrane was the smallest among all the membranes investigated, mostly due to the smooth surface of the LDH itself, as shown in Figure 4a. When the CNTs were incorporated in the membrane at a level of 1 to 2 wt.%, the Rq decreased from 35.8 to 33.6 nm. Comparatively, a continuous addition of CNTs up to 5 wt.% led to an increase in the Rq to 43.9 nm. Such a phenomenon could be rationalized by the agglomeration of CNTs in the composite membrane. The low weight ratio of CNTs (1 wt.%–2 wt.%) facilitated its dispersal in the LDH nanosheets, which weakened the agglomeration phenomenon of the CNTs, while the increasing addition of CNTs (3 wt.%–5 wt.%) contributed to the boosted surface roughness via the agglomeration of CNTs. In summary, the intercalation of CNTs could improve the contact area between the feed liquid and the membrane via the increased membrane surface roughness, obviously leading to an enhancement in the permeability of the fabricated membrane.

### 3.2. Dye Removal Behavior of CNTs/LDH Composite Membranes

**Figure 5:**
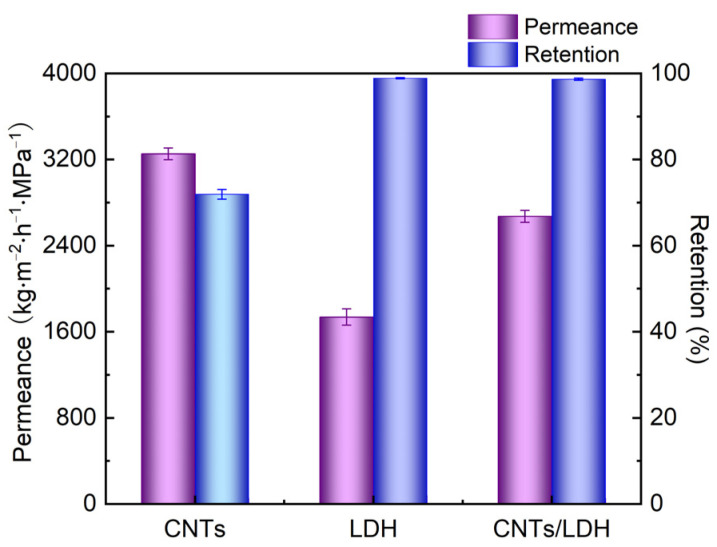
Dye removal performance of CNT, LDH, and CNTs/LDH membranes (operation condition: 0.1 g/L Evans blue aqueous solution; operating pressure: 0.1 MPa).

In order to explore the fabrication conditions of the CNTs/LDH composite membrane, the dye removal performances of the CNT membrane, LDH membrane, and CNTs/LDH membrane were comprehensively investigated. Figure 5 depicts the removal performance of Evans blue for the aforementioned membranes. The permeance of the CNT membrane reached 3200 kg/(m^2^·h·MPa), while the corresponding retention was only 71%. This behavior is ascribed to the characteristics of CNTs. As a 1D nanomaterial, the deposition of CNTs on hydrolyzed PAN membrane failed to form a compact separation layer on the substrate, as demonstrated in Figure 3a. In terms of the LDH membrane, there was no pore on the separation layer surface and water could only go through the membrane via the interlayer channels. Although dissolved CO_2_ in water facilitates the intercalation of CO_3_^2−^ in the exfoliated LDH nanosheets to form possible water channels, the accumulation of LDH without intercalation is dominant in the LDH membrane, resulting in a relatively compact membrane surface. Consequently, the permeance reduced to 1700 kg/(m^2^·h·MPa) with a retention of 98.8% due to the compact structure. When CNTs were intercalated into LDH to form a composite membrane, the permeance increased to 2600 kg/(m^2^·h·MPa), which was just between that of the CNT membrane and the LDH membrane. Additionally, the retention was improved to 99%, clearly verifying the synergistic effect between CNT and LDH. Specifically, the increased permeance compared with the LDH membrane verifies the successful intercalation of CNTs into LDH, while the high retention confirms that a separation layer without obvious defects is formed. Therefore, the comparison between these three membranes confirms the interaction between the compact membrane structure and the layer spacing, which alleviates the resistance to water transport and leads to the rejection of dye molecules by a separation layer without obvious defects.

**Figure 6:**
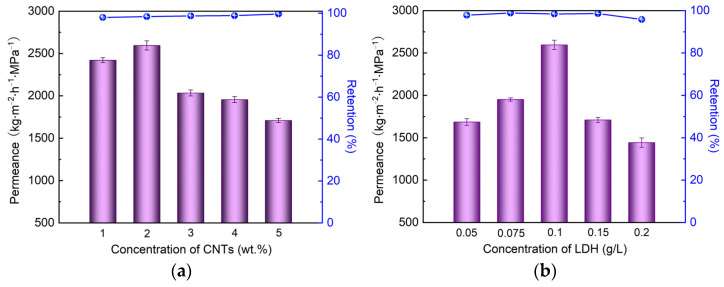
(**a**) The effects of CNTs concentrations on the separation performance (operation condition: 0.1 g/L Evans blue aqueous solution; operating pressure: 0.1 MPa); (**b**) the effects of LDH concentrations on separation performance (operation condition: 0.1 g/L Evans blue aqueous solution; operating pressure: 0.1 MPa).

Next, the weight ratio of CNTs in the composite membrane was investigated, as the addition of CNTs alters the permeance as well as the retention of the obtained membranes. As illustrated in Figure 6a, the permeance climbed from 2400 to 2600 kg/(m^2^·h·MPa) with the addition of CNTs at a level of 1 wt.% to 2 wt.%, which apparently contributed to the significantly increase in the number of interlayer water transport channels via the accumulation of CNT intercalation into LDH nanosheets. Moreover, water molecule could pass through the CNT channels rapidly via the capillary force of the CNT channels, obviously resulting in the promotion of permeance [43,44]. Nevertheless, the permeance decreased to 1710 kg/(m^2^·h·MPa) with the addition of CNTs up to 5 wt.%. This fading trend of permeance is attributed to the excess amount of CNTs in the composite membrane. Specifically, CNTs were in great excess in the intercalation into LDH, which led to the aggregation of CNTs. This aggregation phenomenon would inevitably increase the mass transport resistance of the resultant membrane and the corresponding permeance would be decreased. Based on the results above, the weight ratio of CNTs was fixed at 2 wt.% for the fabrication of the CNTs/LDH membrane.

Besides the amount of CNTs in the composite membrane, the concentration of LDH solution is crucial to the separation performance of the membrane. Figure 6b depicts the influence of the concentration of the LDH solution on the filtration performance of the obtained membranes. The permeance of the obtained membrane increased from 1685 to 2600 kg/(m^2^·h·MPa) along with the increase in LDH concentration from 0.05 to 0.1 g/L. This growth of permeance can again be ascribed to the relative addition ratio between LDH and CNTs in the composite membrane. The amount of LDH was not sufficient and CNTs were in excess in the fabricated membrane. Thus, the aggregation phenomenon dominated the filtration performance of the membrane when the LDH concentration was 0.05 g/L. When the LDH concentration was raised continuously to 0.2 g/L, the corresponding permeance decreased to 1440 kg/(m^2^·h·MPa). Under such circumstances, the CNTs were not sufficient for the construction of a mass transport channel of CNTs/LDH, causing the exploited LDH nanosheets themselves to build up the transport channel. Hence, a decreased permeance of the membrane was observed mainly due to the increase in the mass transport resistance. Given the above results, the optimal LDH concentration was kept at 0.1 g/L.

**Figure 7:**
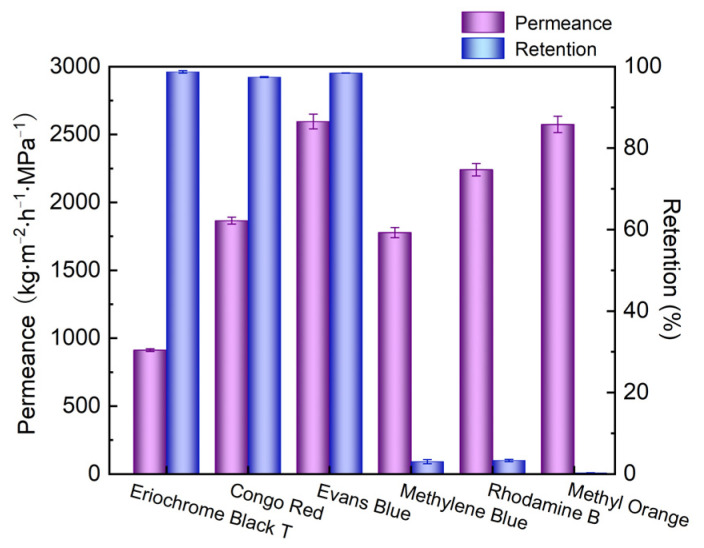
Separation performance of the CNTs/LDH membrane for different dyes (preparation conditions: 0.1 g/L LDH, 2 wt.% CNT; operating pressure: 0.1 MPa).

Based on the optimal conditions mentioned above, the fabricated CNTs/LDH membrane was used to evaluate the separation performance of six dyes, including EBT, CR, EB, RhB, MB, and MO, as demonstrated in Figure 7. Clearly, the retentions for EBT, CR, and EB were about 98%, while the retentions for the other three dyes were below 5%. This difference mainly stemmed from the interaction between the dye and the membrane. As demonstrated in Table 1, the molecular sizes of EBT and RhB were similar. However, the retention of EBT was superior to that of RhB, confirming that the Donnan effect was the dominant factor in the determination of the filtration performance. Specifically, CNTs in the interlayer of the CNTs/LDH composite membrane were negatively charged and anionic dyes were thus repelled by the intercalation molecule. Moreover, both cationic and zwitterionic dyes employed in this study were attracted by CNTs in the membrane; thus, the retention of both types of dyes was neglected. Among all dyes, the fabricated membrane possessed the highest permeance (2600 kg/(m^2^·h·MPa)) and retention (98%) for EB.

**Figure 8:**
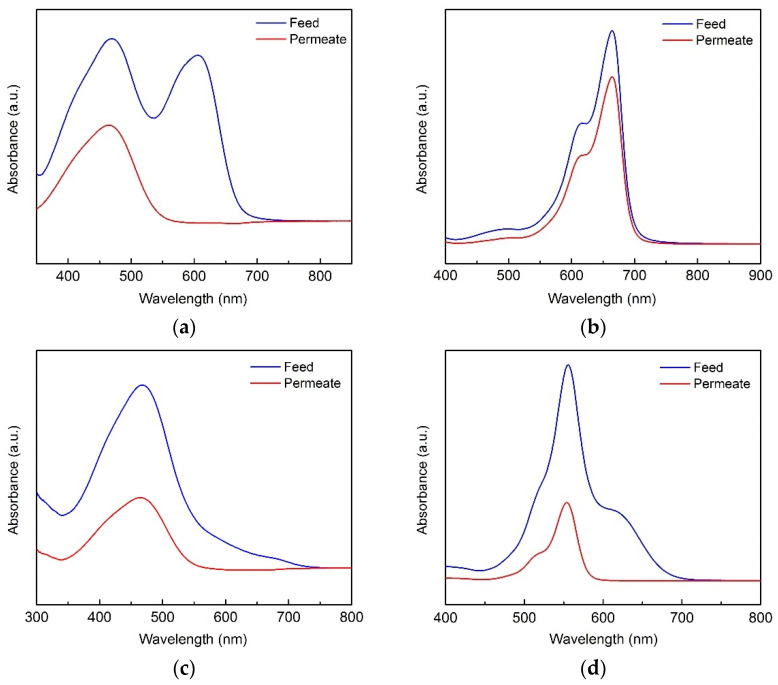
Absorbance spectra of the feed and permeate with binary dye mixtures of (**a**) EB/MO, (**b**) MB/CR, (**c**) EBT/MO, and (**d**) RhB/EB (preparation conditions: 0.1 g/L CoAl-LDH, 2 wt.% CNT; operating pressure: 0.1 MPa).

**Figure 9:**
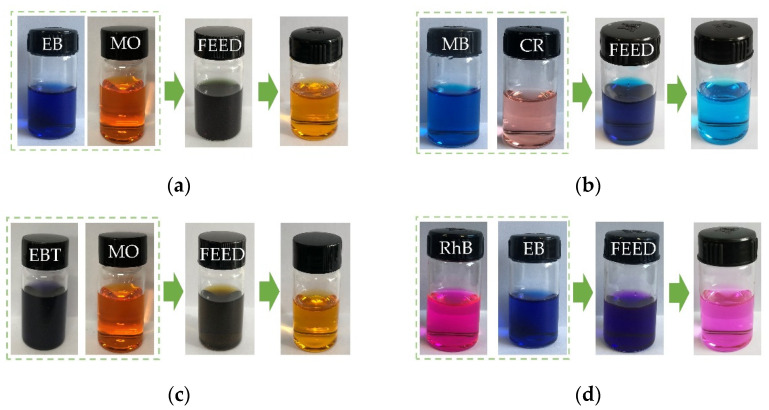
Samples of feed and permeate with binary dye mixtures of (**a**) EB/MO, (**b**) MB/CR, (**c**) EBT/MO, and (**d**) RhB/EB.

Considering the specific sieving mechanism of the proposed membrane, dye mixtures were employed to further evaluate the filtration performance. As shown in Figure 8 and Figure 9, four groups of binary dye mixtures were selected and the corresponding UV–vis absorption spectrum and permeance were demonstrated. The absorption peaks of EBT, CR, EB, RhB, MB, and MO were at 335, 497, 610, 550, 662, and 463 nm, respectively. When EB was mixed with MO under a weight ratio of 1:1, the binary dye mixtures were dark cyan in color. After filtration, the permeate was orange, which was the color of the MO. Such a phenomenon agrees well with the single dye filtration test, as shown in Figure 7, clearly verifying that the separation mechanism was dominated by the Donnan effect.

Additionally, a fading trend of the orange color was observed, which was apparently fulfilled by the electrostatic interaction between negatively charged EB and positively charged MO. Such an interaction resulted in the aggregation of dyes, meaning that the corresponding retention of MO was increased. The retention of EB was also verified by the UV–vis absorption spectrum shown in Figure 8a. Two evident absorption peaks at 463 and 610 nm, representing the existence of MO and EB, were demonstrated before the filtration, while only one absorption peak at 463 nm was kept. Additionally, the decrease in peak absorbance confirmed the increase in MO retention. The situation of the other three binary mixtures was similar to that of EB and MO. Noteworthily, as a zwitterionic dye, RhB could permeate through the composite membrane and a weakened absorption peak at 550 nm was shown after filtration in Figure 8d, thus verifying the attraction between CNTs and zwitterionic dye.

### 3.3. Humic Acid Removal Performance of CNTs/LDH Composite Membranes

**Figure 10:**
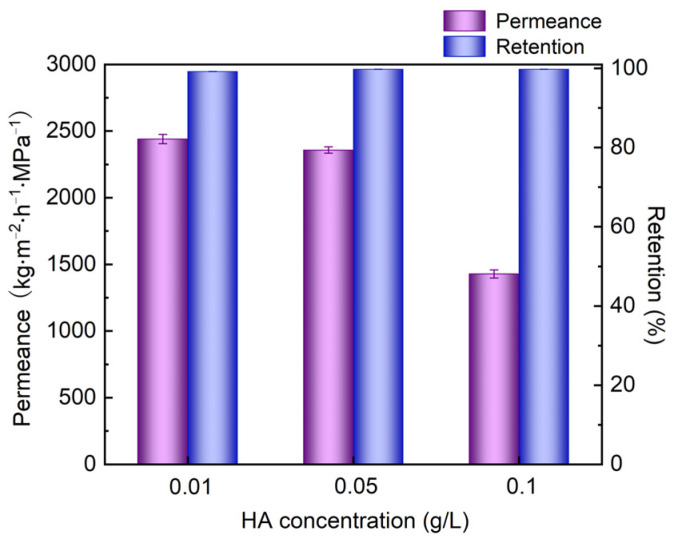
Effect of HA concentration on the separation performance of the CNTs/LDH membrane (preparation conditions: 0.1 g/L LDH, 2 wt.% CNT; operating pressure: 0.1 MPa).

**Figure 11:**
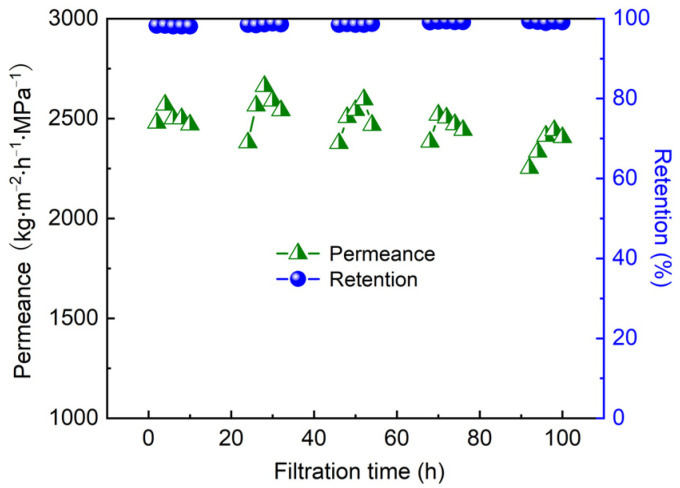
Stability test of CNTs/LDH composite membrane for HA removal (preparation conditions: 0.1 g/L LDH, 2 wt.% CNT; operating pressure: 0.1 MPa).

As one of the main organic foulants in wastewater treatment, humic acid (HA) influences the quality of drinking water [45,46]. Thus, it is crucial to investigate the filtration performance of HA. Figure 10 demonstrates the permeance and retention rate of CNTs/LDH composite membranes for different concentrations of HA scoping from 0.01 to 0.1 g/L. Initially, a permeance of 2400 kg/(m^2^·h·MPa) with a retention of 99% was revealed when the HA concentration was 0.01 g/L. Such a high retention stemmed from the negatively charged molecules on HA, which were repelled with CNTs in the fabricated membrane. Moreover, the permeance and retention rate stayed almost constant along with the increase in HA concentration up to 0.05 g/L, further confirming the applicability of the proposed membrane for HA removal applications. Nevertheless, the permeance decreased to 1428 kg/(m^2^·h·MPa) with an unaltered retention, clearly confirming the existence of an HA filtration cake on the surface of the membrane. Specifically, the permeance of the membrane reduced by 2% and 41% at HA concentrations of 0.05 g/L and 0.1 g/L, respectively. Comparatively, permeance reductions of 33% and 64% were observed at the same HA concentrations in our previous research [42], verifying that the proposed membrane had superior antifouling properties. Furthermore, a long-term stability test was carried out to evaluate the practical application of the membrane in industry. As illustrated in Figure 11, the membrane was tested during the daytime and then soaked in the feed solution overnight (shaded region). After 100 h of the filtration test, no obvious drop in permeance and retention was observed for the CNTs/LDH composite membrane. Therefore, the proposed membrane was capable of the removal of both dye and HA in aqueous solutions. In summary, considering the facile membrane fabrication process as well as the relatively low cost of the raw material of the composite membrane, the CNTs/LDH membrane could be utilized as a robust tool in water purification applications.

## 4. Conclusions

In this paper, a CNT-intercalated CoAl-LDH composite membrane was prepared via vacuum-assisted assembly for use in water purification. CNTs were intercalated in CoAl-LDH nanosheets via electrostatic interaction and the obtained laminates were fabricated to form a composite membrane via vacuum-assisted assembly. With the assistance of the XRD, SEM, and AFM characterization of the membrane morphologies and components, CNTs were observed to be successfully intercalated in LDH nanosheets. Additionally, the obtained nano-channels facilitated the mass transport of water via low flow resistance between CNT and water, thus promoting the permeance of the CNTs/LDH composite membrane. Based on the results above, optimized fabrication conditions were determined (the concentration of CoAl-LDH was 0.1 g/L and the weight ratio of CNTs was 2 wt.%). Among all the tested dyes, the proposed membrane had a superior permeance as well as retention for EB (permeance = 2600 kg/(m^2^·h·MPa), retention = 98%). Moreover, the separation mechanism was discussed depending on the dye molecular size and the charge type in both single and binary dye combinations. It was concluded that the Donnan effect was the dominant factor in the filtration process of the CNTs/LDH composite membrane. Furthermore, a stability test of HA at a filtration pressure of 0.1 MPa was investigated, clearly confirming that the CNTs/LDH composite membrane is promising for use in water purification.

## Figures and Tables

**Table 1:** Molecular information of the experimental dyes.

Dye	Molecular Weight	Molecular Size	Dye Type
EBT	461.4 Da	1.6 nm × 0.9 nm	Anionic
CR	696.7 Da	2.6 nm × 0.7 nm	Anionic
EB	960.8 Da	1.2 nm × 3.1 nm	Anionic
RhB	479 Da	1.2 nm × 1.13 nm	Zwitterionic
MB	319.9 Da	1.3 nm × 0.5 nm	Cationic
MO	327.3 Da	1.47 nm × 0.53 nm	Cationic ^a^

^a^ This type of dye was determined by the absorption spectra of methyl orange (463 nm), since the protonated form of MO could be zwitterionic (507 nm).

## Data Availability

The data presented in this study are available on request from the corresponding author.
